# Providing financial incentives to rural-to-urban tuberculosis migrants in Shanghai: an intervention study

**DOI:** 10.1186/2049-9957-1-9

**Published:** 2012-11-01

**Authors:** Xiaolin Wei, Guanyang Zou, Jia Yin, John Walley, Huaixia Yang, Merav Kliner, Jian Mei

**Affiliations:** 1 School of Public Health and Primary Care, The Chinese University of Hong Kong, Prince of Wales Hospital, Shatin, NT, Hong Kong, China; 2 COMDIS China Program, Nuffield Centre for International Health and Development, University of Leeds, Room 403, No. 1032 Dongmen North Rd, Luohu District, Shenzhen, 518003, China; 3 Nuffield Center for International Health and Development, University of Leeds, 101 Clarendon Rd, Leeds, LS2 9LJ, UK; 4 Shanghai Changning District Center for Disease Control and Prevention, No.39 Yun Wu Shan Rd, Shanghai, China; 5 Shanghai Center for Disease Control and Prevention, No. 1380 Zhongshan Xi Rd, Shanghai, China

**Keywords:** Public health, Tuberculosis, Domestic migrants, Poverty, Financial incentive, Treatment completion, Effectiveness

## Abstract

**Background:**

Financial issues are major barriers for rural-to-urban migrants accessing tuberculosis (TB) care in China. This paper discusses the effectiveness of providing financial incentives to migrant TB patients (with a focus on poor migrants in one district of Shanghai using treatment completion and default rates), the effect of financial incentives in terms of reducing the TB patient cost, and the incremental cost-effectiveness ratio of the intervention.

**Results:**

Ninety and ninety-three migrant TB patients were registered in the intervention and control districts respectively. TB treatment completion rates significantly improved by 11% (from 78% to 89%) in the intervention district, compared with only a 3% increase (from 73% to 76%) in the control district (P = 0.03). Default rates significantly decreased by 11% (from 22% to 11%) in the intervention district, compared with 1% (from 24% to 23%) in the control district (P = 0.03). In the intervention district, the financial subsidy (RMB 1,080/US$170) accounted for 13% of the average patient direct cost (RMB 8,416/US$1,332). Each percent increase in treatment completion costs required an additional RMB 6,550 (US$1,301) and each percent reduction in defaults costs required an additional RMB 5,240 (US$825) in the intervention district.

**Conclusions:**

Overall, financial incentives proved to be effective in improving treatment completion and reducing default rates among migrant TB patients in Shanghai. The results suggest that financial incentives can be effectively utilized as a strategy to enhance case management among migrant TB patients in large cities in China, and this strategy may be applicable to similar international settings.

## Multilingual abstracts

Please see Additional File 1 for translations of the abstract in six official working languages of the United Nations.

## Background

China has the second largest TB burden in the world, with the prevalence of active TB cases in rural areas twice that of urban areas [1]. Increasing population mobility has become one of the major challenges in TB control, especially with more than 200 million people moving from rural areas to more prosperous urban areas in recent years [2]. The Chinese residency permits system is one where each newborn child is assigned a record known as a *hukou*, which is in accordance with the residence status of the child's parents. Local financing of public services is allocated according to the number of *hukou* holders in the particular local area migrants are thus less entitled to the local social securities and public services [3-5]. Migrants often take unstable and low paid jobs in informal sectors such as restaurants and construction sites.

Shanghai is one of the largest and most developed cities in China. In 2010, Shanghai had a population of 23 million, with 9 million internal migrants. Prevention and control of TB within the migrant population is a great challenge – 50% of all TB cases in Shanghai in 2007, for example, were diagnosed in migrants. In 2005, half of the migrant TB patients defaulted from treatment and the cure rate was much lower than that of permanent residents [6].

In Shanghai, TB services are provided by an integrated system consisting of a lung clinic in the designated district general hospitals, the TB dispensary (normally integrated with control services of sexual diseases, HIV/AIDS and leprosy) at the Center for Disease Control (CDC), and the disease prevention departments in the Community Health Centers (CHCs). The lung clinic in the designated hospital provides TB diagnosis and treatment in line with the national TB control guidelines [7]. TB patients, or those suspected to have TB, who have had a cough lasting for more than two weeks are referred to the lung clinic, which then reports the case to the TB dispensary. The TB dispensary is responsible for the public health tasks of TB control, such as staff training and case supervision. At the guidance of the TB dispensary, health workers in the CHCs provide treatment supervision and follow up with patients during the systematic TB case management process known as DOTS (Directly Observed Therapy Short Course).

Free treatment is provided to TB patients who are registered in the TB dispensary regardless of their *hukou* status. The free treatment policy covers the costs of the whole course of first line anti-TB drugs, and basic examinations including sputum smear and culture, chest X-ray, and blood profiles for liver function. Patients have to pay upfront and then claim reimbursement upon completion of the whole treatment. The policy does not cover hospitalization costs, second line anti-TB drugs, and any other drugs or medical examinations. In Shanghai, TB patients pay all medical costs, keep the receipts and are reimbursed all the costs covered by the free treatment policy at the end of their treatment.

Poor adherence to TB treatment can result in treatment failure, generate multidrug resistance (MDR) TB cases, and increase TB transmission in the community [8,9]. Treatment adherence is related to patient's socio-economic factors, health system barriers, and compliance to the treatment regiments [10,11]. In China, poor adherence to TB treatment is associated with heavy financial burden, lack of social support, side effects of drugs, and patient’s mobility [12]. Despite China’s free treatment policy, financial costs were found to be the most cited reasons for TB treatment default [13]. Our qualitative study in Shanghai confirmed that financial barriers were the biggest factors causing default in migrant TB patients [4].

Among the various interventions [14-16], financial incentive is one of the most commonly implemented and evaluated methods to improve TB treatment adherence [17]. Use of financial incentives has proved to be beneficial in TB treatment completion among vulnerable populations such as prisoners [18,19], homeless populations [20-22], and drug users [23-26]. However, most of the experiences have been limited to the prophylactic treatment of latent TB. Although interventions may often target poor TB patients, they do not conduct a systematic process of poverty evaluation, thus compressing the impact for poverty elevation in such programs [27,28].

No studies to date have been published regarding the policy and practices of providing financial incentives to migrant TB patients, especially poor migrants in large cities. In this paper discusses the effectiveness of providing financial incentives to migrant TB patients (with a focus on poor migrants in one district of Shanghai using treatment completion and default rates), the effect of financial incentives in terms of reducing the TB patient cost, and the incremental cost-effectiveness ratio of the intervention.

## Results

### Background characteristics of subjects

During the study period, there were 90 and 93 TB patients registered in the intervention and control districts respectively. In the intervention district, there were 81 (90%) new patients (i.e. patients who had no previous treatment) and there were 82 (88%) new patients in the control district (P > 0.05). No extra-pulmonary cases or severe cases such as coughing with blood were found in migrant TB patients registered in the two districts during the study period. No significant difference was found in gender, average age, marriage status, employment status, education and insurance status of migrant TB patients between the two districts (P > 0.05, see Table 1). Based on the questionnaire undertaken with participants, 70 (78%) migrant TB patients in the intervention district and 56 (60%) migrant TB patients in the control district were assessed as living in poverty. Of the poor 136 migrant TB patients in the intervention district, 46 (66%) received the subsidy of RMB 1,000; while 80 (89%) of all the migrant TB patients received the transport incentives of RMB 80. Among the 24 (34%) poor patients who did not receive the financial subsidy, 20 (83%) declined to receive it, while 4 (17%) returned to their hometowns upon poverty assessment.

**Table 1:** Demographics of migrant TB patients in the intervention and control districts

**Variables**	**Intervention**	**Control**
Subjects	90	93
Male (%)	48 (53)	58 (62)^a^
Average age	30	34^b^
Married (%)	44 (49)	51 (55)^c^
Education: illiterate and semi-literate (%)	10 (11)	12 (13)^d^
Employment: informal sector (labor intensive and service industry) (%)	70 (78)	81 (87)^e^
Have medical-related insurance in Shanghai (%)	14 (16)	21 (23) ^f^

^a^χ^2^ = 1.531,P = 0.216;^b^Z = 0.759,P = 0,448;^c^χ^2^ = 0.649,P = 0.421;^d^χ^2^ = 0.139;P = 0.709;^e^χ^2^ = 2.753, P = 0.097;^f^χ^2^ = 1.459, P = 0.227.

### Treatment completion rates

**Figure 1:**
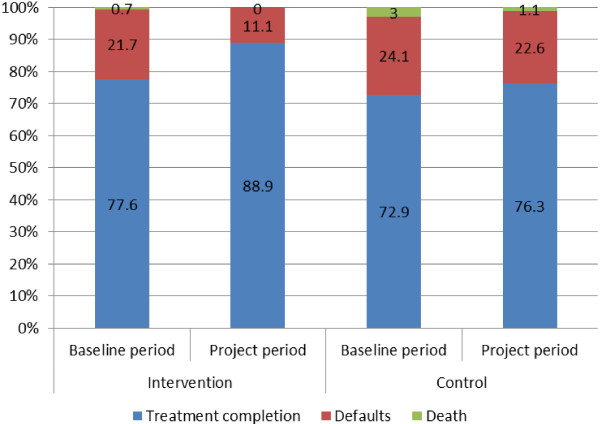
Treatment outcomes of migrant TB patients in the intervention and control districts.

Compared with baseline data, treatment completion rates of migrant TB patients improved in both districts during the study period (see Figure 1). A statistically significant increase of 11% in treatment completion rate was observed in the intervention district (78% to 89%, *χ*2 = 4.745, P = 0.03), while no significant increase was observed in the control district (73% to 76%, P = 0.56). The increase of treatment completion rates in the intervention district was significantly higher than that in the control district (11% vs. 3%, p = 0.03).

### Default rates

A total of 10 migrant patients defaulted in the intervention district, with 7 (70%) of these defaulting within the first month of treatment. A similar situation was reported in the control district, with 13 (62%) defaulting in the same period. Both districts observed reduced default rates of all the migrant TB patients after the intervention as compared with the baseline data (see Figure 1). The reduction in default rates was statistically significant in the intervention district (22% to 11%, *χ*^2^ = 4.254, P = 0.04), but not in the control district (24% to 23%, P = 0.80). The reduction of default rates of all patients in the intervention districts was significantly larger compared with that of the control district (11% vs. 1%, P = 0.03).

### Effect of financial incentives

The number of patients who completed their treatment was 73 (90%) and 65 (79%) of new migrant TB patients in the intervention and control districts respectively. Of these, 52 (71%) of the patients in the intervention district and 43 (66%) in the control district completed the questionnaire about their financial burdens (see Table 2). This relatively low response rate was likely due to the mobile nature of migrants. The proportion of direct cost accounted for 16% and 18% of the average annual family income in the intervention and control districts respectively. In the intervention district, the financial subsidy (RMB 1,080/US$170) accounted for 13% of the average patient direct cost (RMB 8,416/US$1,332). The transportation incentive (RMB 80/US$13) over the treatment period accounted for 28% of the patients' average transportation costs (RMB 290/US$46).

**Table 2:** Cost of treatment for new TB patients in the intervention and control districts

**Variables**	**Intervention**	**Control**
No of subjects	52	43
Male (%)	31 (60)	25 (58)^a^
Average age	30	35^b^
Married (%)	25 (48)	13 (30)^c^
Education: illiterate and semi-literate (%)	3 (5.8)	5 (12)^d^
Employment: informal sector (labor intensive and service industry) (%)	35 (67)	35 (81)^e^
Have medical insurance in Shanghai (%)	9 (17)	10 (23)^f^
Average per capita annual family income (RMB)	27,194	22,124^g^
Average annual family income (RMB)	53,004	53,605^h^
Direct cost (RMB)	8416	9743^i^
Direct medical cost (RMB)	5929	6902^j^
Direct non-medical (RMB)	2487	2841^k^
Transportation cost (RMB)	290	262^l^
Food and accommodation (RMB)	246	485^m^
Nutrition cost (RMB)	1950	2094^n^
Direct cost/annual family income (%)	16	18

^a^χ^2^ = 0.021, P = 0.884; ^b^Z = −1.277, P = 0.202;^c^χ^2^ = 3.123, P = 0.077;^d^Fisher’s exact test, P = 0.461;^e^*χ*^2^ = 2.409, P = 0.121;^f^χ^2^ = 0.520, P = 0.471;^g^Z = −0.969, P = 0.333;^h^Z = −0.565, P = 0.572;^i^ Z = −1.436, P = 0.151;^j^Z = −1.028, P = 0.304; ^k^Z = −1.537, P = 0.124; ^l^NZ = −0.345, P = 0.730; ^m^Z = −0.260, P = 0.795; ^n^Z = −0.931, P = 0.352.

### Incremental cost-effectiveness analysis

In total, this project involved an investment of an additional RMB 52,400, which consisted of RMB 46,000 of financial subsidy and RMB 6,400 of transport incentives. This additional cost prompted an increase of 8% in treatment completion rate in the intervention district as compared to the control district. This suggests that for each percent increase in treatment completion, an additional cost of RMB 6,550 (US$1301) was invested in the intervention district. Similarly, this additional cost delivered a reduction of 10% in the default rate in the intervention district compared with the control district, showing that an additional cost of RMB 5,240 (US$825) was needed to reduce each percent in default rates (see Table 3).

**Table 3:** Incremental cost-effectiveness of the intervention

	**Intervention**	**Control**
Incremental cost (RMB)	52,400	0
Treatment completion rate:		
Baseline period (%)	78	73
Project period (%)	89	76
Incremental effect (%)	11	3
Net incremental effect (%)	8	-
Incremental cost per increase of 1% treatment completion, when compared to the control arm (RMB)	6,550	
Default rate:		
Baseline period (%)	22	24
Project period (%)	11	23
Incremental effect (%)	11	1
Net incremental effect (%)	10	-
Incremental cost per decrease of 1% default rate, when compared to the control arm (RMB)	5240	

## Discussion

Our study showed that providing financial incentives to migrant TB patients was effective in improving treatment completion and reducing default rates. Our early qualitative study found that financial constraints were substantial barriers to the provision of TB services among migrant patients in Shanghai [4]. This study also suggests that providing financial incentives reduces the access barrier for migrants and contributes to treatment completion. In the intervention district, the financial assistance provided to migrants accounted for a significant proportion of their medical costs, transportation costs, nutrition and food, and accommodation costs associated with treatment, and was especially beneficial for the poor patients. Tulsky et al. suggests that cash incentives, such as the ones used in this study, may be more effective than non-cash incentives in improving treatment adherence [21]. However, cash incentives also have limitations. Patients may use this to purchase items such as alcohol and cigarettes, which may in turn inadvertently affect treatment results [29,30]. However, we did not find evidence of this in our study.

Our study also found that providing the financial subsidy in four installments during the course of the treatment was effective as it appeared to motivate patients to complete their treatment. Another study found that monetary incentives were effective in improving adherence to TB treatment, however, this was limited to the first follow-up appointment in homeless individuals only [22]. Davidson et al. found that the improved adherence to TB treatment was attributed to the financial incentives which were distributed on a progressive and cyclical basis with a bonus at the end of each cycle period [31]. Although Davidson’s model may be a better way to motivate the completion of treatment, it is too complicated to apply in routine busy practice. In our study, the monetary incentives, working as performance rewards [32], were given to migrants in stages when they had to renew their drugs. This was proved to be just as effective as Davidson’s model.

Our study found that, in both districts, the majority of the migrant TB patients were living in poverty. In the intervention district, the majority of the poor patients received a financial subsidy of RMB 1,000. The study proved that our simple process of poverty evaluation is feasible and patient-friendly, which is an important step in ensuring the appropriate distribution of financial incentives [27,28]. Our project suggests that it is feasible to engage community-based health workers, especially as they are closer to the migrant residents and have the responsibility of following up with TB patients.

In this study, 11% of the patients in the intervention district defaulted from treatment, while a large proportion of patients defaulted within one month of registration despite the incentives. This may be due partially to a delay in conducting the poverty assessment and distributing the financial incentives. This again highlights the importance of timely and appropriate administration of financial incentives for the scheme to be successful [27,31]. However, given the higher living costs in Shanghai and the lack of family support for migrants, the financial subsidy may not be sufficient to attract patients to stay in the city to complete their treatment. Our earlier study showed that patients who did not own any property in Shanghai, and/or were unemployed because of TB, were likely to return to their hometown upon diagnosis for better family support and care [4]. The post-reimbursement procedures of TB treatment in Shanghai and the profit-seeking behaviors of public hospitals may also add greater financial burdens for TB patients during their treatment [4].

Our study found that an additional cost of RMB 6,550 (US$1301) is needed in the intervention district to achieve a 1% increase of treatment completion, and an additional cost of RMB 5,240 (US$825) is needed for a 1% decrease of default rates as compared to the control district. If these financial incentives are to continue, the government will be required to provide this additional funding. However, given the financial capacity of Shanghai and other similar cities, as these costs are relatively small, they should not pose an obstacle to reducing TB transmission in migrants and local residents.

This project was designed to be embedded in existing TB services, that is, no extra staff time was required in TB clinics and CDCs. In the meantime, a similar project funded by the Global Fund to Fight Against AIDS, Tuberculosis and Malaria (GFATM) was implemented in Shanghai. This project has increased clinical hours and appointed new staff, in addition to providing financial incentives to migrant TB patients. Further economic evaluation is being conducted to compare the effects of two different ways of providing financial incentives to migrant TB patients elsewhere.

China is building a harmonious equitable society. Our study may contribute to a policy change for providing the general social protection system for migrants in Shanghai and other large cities in China. Various government sectors, such as health insurance, civil affairs, and TB control can be mobilized to replicate this program.

This study had a few limitations. Firstly, it had limited generalizability as it was conducted in one big city, resulting in a test pool of a relatively limited number of migrant TB patients. Nevertheless, the study has detected a significantly statistical difference on treatment completion rates and default rates, and therefore this may provide an insight for migrant TB control in similar settings. Secondly, nearly a quarter of the poor patients did not receive the financial subsidy in the intervention district. This may be due partially to program administration problems and to the fact that poor patients were reluctant to receive public support. Thirdly, our study was conducted in the context of the increasingly improved TB control efforts and outcomes. However, the improvement effect can be largely balanced by comparing the control and intervention districts during the same period. To our knowledge, no other policies or economic changes could be taken into account that may influence the treatment of the migrant TB patients at that time. Finally, due to the mobile nature of migrants, only around 70% of the new migrant TB patients completing treatment were able to complete the questionnaire about their financial burdens. Our study may be subject to recall bias as patient direct non-medical costs were collected from patient surveys as no other methods of data collection were available. However, patient direct medical costs in TB clinics were collected from patient charts review. Furthermore, the study was conducted shortly after patients' treatment completion to minimize recall bias.

## Conclusions

Overall, the study can conclude that financial incentives proved to be effective in improving treatment completion and reducing default rates among the migrant TB patients in Shanghai. The results suggest that financial incentives can be effectively utilized as a strategy to enhance case management among migrant TB patients in large cities in China and this may be applicable to similar international settings.

## Methods

### Interventions

A controlled before-and-after design was employed to investigate the effectiveness of financial incentives on treatment completion and default rates among all the migrant TB patients. The intervention commenced in October 2007 and was conducted in one district of Shanghai. In the intervention district, each migrant TB patient received RMB 10 (US$2) (1US$ = 6.35 RMB) per month for transportation to encourage them to visit the TB clinic in the designated hospital. In addition, a living subsidy of RMB 1,000 (US$157) was provided to poor migrant TB patients. The intervention was designed to fit into the routine practices and job descriptions of the health providers from the CDC, TB clinics in the designated hospitals, and CHCs. The migrant TB patients were informed of the availability of the financial incentives upon diagnosis in the TB clinics. The trained health workers from the CHCs met the migrant TB patients upon registration, assessed their poverty status (using a standard questionnaire), and provided the financial incentives to them. Each community has a team of community health workers, including one or two general practitioners and three to four nurses. The team provides integrated primary health care and public health services to the residents in the communities. The salaries of community health workers are fully provided by the government. Community health teams have the responsibility of taking care of their catchment population and carrying out routine check-ups at patient homes. Community health workers usually have a good knowledge of local communities and know the local residents. Standard TB treatment includes an initial intensive phase (to kill the actively growing bacilli, lasting 2 months), and a continuation phase (to sterilize by destroying all the bacilli, lasting 4 months for newly treated cases and 6 months for retreated cases) [7]. According to the current requirements, community health workers visit TB patients, regardless of their residency status, twice a month in the intensive phase and once a month in the continuation phase (the subsequent four months of standard TB treatment for newly treated cases, and 6 months for retreated cases). Therefore, community health workers are the most appropriate people to assist in the intervention process.

**Figure 2:**
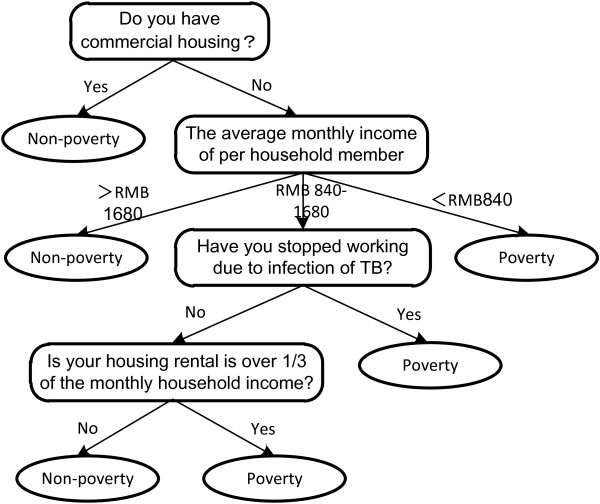
The poverty assessment tool for migrant TB patients.

Based on the results of our exploratory study [4], migrant TB patients who owned a commercial property in Shanghai or whose family earned twice the minimum level of income set by the Shanghai Government in 2005 [33], were classified as non-poor. Migrant TB patients who did not own a property and earned less than the Shanghai minimum income were defined as poor. Migrant TB patients who did not have a property and earned less than twice but higher than the Shanghai minimum income were asked questions regarding whether they were unemployed due to the disease or if their rent accounted for more than a third of their family income. Migrant TB patients who answered yes to any of these questions were defined as poor (see Figure 2). The subsidy was given in four cash installments: RMB 300 (US$47) at the time of diagnosis, RMB 300 (US$47) at the end of the second month of treatment, RMB 200 (US$31) at the end of the fourth month of treatment, and RMB200 (US$31) at the end of treatment.

Another district was selected as the control district, where patients were given the usual care and no financial incentives were given specifically for migrant patients whose poverty status was also assessed. In both districts, the TB DOTS program (encompassing the standard and free treatment of TB as described previously) was applied to all TB patients, including the migrants. District names were made anonymous to protect their identities.

### Evaluation

Data were collected through routine TB registers in both districts for patients who were enrolled at two 15-month time periods, i.e. the baseline period of 1 July 2006 to 30 September 2007 and the study period of 1 October 2007 to 31 December 2008. All the migrant TB patients who were diagnosed in the intervention and control districts during the above periods were registered regardless of whether the treatment had begun or not. To evaluate the effectiveness of the intervention, treatment completion rate was used as the primary outcome and default rate was used as the secondary outcome. Treatment completion rate is defined as the proportion of migrant TB patients who have successfully completed treatment among all the migrant TB patients, including smear positive and negative patients, new patients and retreated patients, who registered during the study period. The default rate is defined as the proportion of migrant TB patients who defaulted from treatment (i.e. in the beginning or during any stage of the treatment) among all migrant TB patients registered during the study period. Definitions regarding treatment completion rates and default rates are in line with the international and national guidelines [7,34]. It is hypothesized that providing financial incentives improves TB treatment completion and reduces defaults among the migrant TB patients in the intervention district compared with the control district. A variance in difference analysis was conducted regarding the increase of treatment completion rates and reduction of default rates between the two districts.

The proportion of MDR TB is usually used as an indicator for case mix of TB patients. However, drug sensitivity analysis was not routinely conducted for TB patients at the time of research. As the retreated cases had a much higher chance of being MDR cases [35], we compared the proportion of new patients as an indicator of case mix in the two study districts.

In the fall of 2009, a face-to-face questionnaire survey to investigate the cost of the TB treatment in both districts was conducted with migrant TB patients who had not been treated previously and enrolled in the two districts during the project period. Patient direct medical costs in TB clinics were collected from medical charts, while direct non-medical costs including transportation, food, accommodation, and nutrition associated with the TB treatment were collected from patient recalls. The aim of the costing analysis was to help understand the financial burden of the migrant TB patients and the effect of the financial incentives in terms of relieving their financial burdens.

A facility survey was conducted to collect the project cost from the CDC. This included the financial subsidy received by the poor TB patients and transportation incentives received by all the TB patients. The incremental cost-effectiveness ratio was calculated to analyze, from the project perspective, the additional cost of having one more percent of patients completing treatment or reducing one percent of patients defaulting from treatment.

Data were analyzed using SPSS 14.0 (USA). Statistical analysis for the primary outcome employed a generalized linear model fitted with a binomial outcome and logit link function, permitting an effect for the district and for the intervention. Data were analyzed using intention to treat analysis. The effect of financial incentives in terms of reducing the TB patient cost was estimated and so was the incremental cost-effectiveness ratio of the intervention:

Incremental cost effectiveness ratio = additional cost of intervention/[increase of treatment completion rate (reduction of default rates) in the intervention district-increase of treatment completion rate (reduction of default rates) in the control district]

Ethical approval for this study was attained from Shanghai Center for Disease Control and Prevention. Informed consent was obtained from all patients.

## Competing interests

The authors declare that they have no competing interests.

## Authors’ contributions

XW, JW, HY, and JM were involved in the conception and design of this project, while XW, GZ, and JY were involved in the implementation of the project and analysis and interpretation of the data. XW and GZ have drafted the manuscripts, while JW and MK have provided critical comments. All authors read and approved the final manuscript.

## Supplementary Material

Additional file 1 Multilingual abstracts in the six official working languages of the United Nations. Click here for file
