# Supplementary material for: Providing financial incentives to rural-to-urban tuberculosis migrants in Shanghai: an intervention study
Source: Infect Dis Poverty. 2012 Nov 1;1:9. doi: 10.1186/2049-9957-1-9 (PMC3710084; doi:10.1186/2049-9957-1-9)
Supplement: Additional file 1 — Multilingual abstracts in the six official working languages of the United Nations. [file 2049-9957-1-9-S1.pdf]

Translation of the abstract into the six official working languages of the United Nations

توفير حوافز مادية للمهاجرين من الريف الى المدينة المصابين بمرض السل في شنغهاي: تجربة علاجية.

كزياولين وي، غوانيانغ زو، جيا ين، جون والي، هوايكسيا يانغ، ميراف كلينر، جيان مي.

خلاصة

معلومات أساسية: تشكل القضايا المالية حاجزاً رئيسياً للمهاجرين من الريف الى المدينة للحصول على الرعاية ضد مرض السل. تفيد هذه الورقة عن فعالية توفير الحوافز المالية للمهاجرين المرضى بالسل، خصوصاً الفقراء منهم، في ما يتعلق بإتمام المعالجة والنسب الرئيسية في منطقة واحدة في شنغهاي، وأثر الحوافز المالية من ناحية تخفيض كلفة المريض بالسل ونسبة فعالية الكلفة المتزايدة للتدخل.

النتائج: جرى تسجيل 90 مهاجراً مصاباً بالسل في منطقة التدخل و93 مهاجراً مصاباً بالسل في منطقة المراقبة. لقد تحسنت نسب إتمام المعالجة ضد السل بنسبة 11% (من 78 الى 98%) في منطقة التدخل، مقارنة بزيادة بنسبة 3% (من 73 الى 76%) في منطقة المراقبة (مؤشر= 0،03). وتراجعت النسب الأساسية بنسبة 11% (من 22 الى 11%) في منطقة التدخل، مقارنة بنسبة 1% (من 24 الى 23%) في منطقة المراقبة (مؤشر= 0،03). وفي منطقة التدخل، احتسبت الإعانة المالية (1،080 رينمبياً/170 دولاراً أميركياً) بنسبة 13% من معدّل كلفة المريض المباشرة (8،416 رينمبياً/1،332 دولاراً أميركياً). وكل زيادة نسبة مئوية في إتمام المعالجة تكلّف زيادة إضافية بقيمة 6،550 رينمنبياً (1301 دولار)، أو كل انخفاض نسبة مئوية في النسب الأساسية يكلّف زيادة إضافية بقيمة 5،240 رينمبياً (825 دلاراً أميركياً) في منطقة التدخل.

الاستنتاج: عموماً، أثبتت التدخلات المالية فعاليتها في تحسين إتمام المعالجة وتخفيض النسب الأساسية بين المهاجرين المرضى بالسل في شنغهاي. تشير النتائج الى ان الحوافز المالية يمكن استخدامها في الواقع كاستراتيجية من أجل تحسين إدارة الحالات الإفرادية بين المهاجرين المرضى بالسل في المدن الكبرى في الصين، ويمكن تطبيق هذه الاستراتيجية في أطر دولية مشابهة.

Translated from English version into Arabic by Liliane Hatem, through

TRANSLATORS WITHOUT BORDERS

上海流动人口结核病患者经济补助干预研究

魏晓林，邹冠炀，阴佳，John Walley，杨怀霞，Merav Kliner，梅建

摘要

背景：在中国，经济困难是流动人口结核病患者就医的主要障碍。本文旨在报告上海某区提供经济补助给流动人口结核病患者，特别是贫困流动人口结核病患者的干预研究，评价项目对治疗完成率和丢失率的影响，经济补助对减轻结核病患者经济负担的作用，以及干预的增量成本效果比率。

结果：项目期间，干预组和对照组分别有 90 和 93 名登记的流动人口结核病患者。与基线数据相比，干预组完成治疗率提高了 11%（从 78%到 89%），而对照组仅提高了 3%（从 73%到 76%）(P=0.03)。干预组丢失率下降了 11%（从 22%到 11%），而对照组仅下降了 1%（从 24%到 23%）(P=0.03)。干预组提供的人均经济补助 1,080 人民币（折合 170 美元)占结核病患者平均直接花费(人民币 8,416 元，折合 1,332 美元，含结核病患者治疗、交通、食宿、营养费).的 13%。平均每增加 1%的完成治疗率需额外投入人民币 6,550 元（折合 1,301 美元），每降低 1%的丢失率需额外投入人民币 5,240 元（折合 825 美元）。

结论：研究表明，经济补助有助于提高上海流动口结核病患者的完成治疗率，降低丢失率。经济补助可以作为中国大城市和其它类似国家和地区加强流动人口结核病病例管理的有效措施。

Translated from English version into Chinese by Zou Guan-Yang

Fournir des incitations financières aux immigrants des zones rurales atteints de tuberculose à Shanghai : une étude d'intervention

Xiaolin Wei, Guanyang Zou, Jia Yin, John Walley, Huaixia Yang, Merav Kliner, Jian Mei

Résumé

Contexte : En Chine, les problèmes financiers sont une barrière majeure pour les personnes migrants des zones rurales vers les centres urbains ayant accès aux soins contre la tuberculose. Cet article rend compte de l'efficacité de fournir des incitations financières aux patients immigrés atteints de tuberculose, en particulier aux immigrants pauvres pour la fin de traitement et le défaut de remboursement dans un district de Shanghai, de l'efficacité des incitations financières en termes de réduction des coûts pour le patient atteint de tuberculose et du taux de rentabilité progressif de l'intervention.

Résultats : Respectivement, 90 et 93 patients immigrés atteints de tuberculose ont été enregistrés dans les districts d'intervention et de contrôle. Les taux d'achèvement du traitement de la tuberculose ont considérablement augmenté de 11% (de 78 à 89 %) dans le district d'intervention, par rapport aux 3 % (de 73 à 76 %) d'augmentation du district de contrôle (P = 0,03). Les taux de défaut de remboursement ont considérablement diminués de 11 % (de 22 à 11 %) dans le district d'intervention, par rapport à 1 % (de 24 à 23 %) dans le district de contrôle (P = 0,03). Dans le district d'intervention, la subvention financière (1 080 RMB / 170 $ US / 130 €) a justifié 13 % du coût direct d'un patient moyen (8 416 RMB / 1 332 $ US / 1 221 €). Chaque augmentation du pourcentage d'achèvement du traitement coûte 6 550 RMB (1 301 $ US / 997 €) supplémentaires – ou chaque réduction du pourcentage de non-remboursement coûte 5 240 RMB (825 $ US / 632 €) supplémentaires dans le district d'intervention.

Conclusion: En général, les incitations financières se sont avérées efficaces pour améliorer l'achèvement du traitement et pour réduire les taux de défaut de remboursement parmi les patients atteints de tuberculose migrants à Shanghai. Les résultats indiquent que les incitations financières peuvent être utilisées avec efficacité comme stratégie pour améliorer la gestion des cas parmi les patients

immigrés atteints de tuberculose dans les grandes villes chinoises, et cela pourrait être appliqué à des situations internationales similaires.

Translated from English version into French by Claire Marie, through

TRANSLATORS
WITHOUT BORDERS

Предоставление в Шанхае финансовой помощи мигрирующим в города больным туберкулезом сельским жителям: интервенционное исследование

Сяолинь Вэй, Гуаньян Цзоу, Цзя Инь, Джон Уолли, Хуайся Ян, Мерав Клинер, Цзянь Мэй

Реферат

Общая информация: Для переселяющихся в города жителей сельских районов Китая финансовые проблемы являются основным препятствием при лечении туберкулеза. В данной статье обсуждается эффективность предоставления финансовой помощи больным туберкулезом мигрантам, особенно из числа бедного населения, для завершения лечения, ее влияние на количество неудачных исходов лечения в одном из районов Шанхая, снижение стоимости терапии больных туберкулезом, а также коэффициент эффективности дополнительных затрат при оказании такой помощи.

Результаты: В районе, где предоставлялась помощь, и в контрольном районе было зарегистрировано 90 и 93 мигранта, больных туберкулезом, соответственно. По сравнению с увеличением на 3% (с 73 до 76%) в контрольном районе, число пациентов, завершивших лечение туберкулеза, значительно возросло (на 11%, с 78 до 89%) в районе, где предоставлялась помощь (P = 0,03). Доля неудачных исходов лечения значительно снизились (на 11%, с 22 до 11%) в районе, где предоставлялась помощь, по сравнению со снижением на 1% (с 24 до 23%) в контрольном районе (P = 0,03). В районе, где предоставлялась помощь, дочерняя финансовая компания оплачивала 13% (1 080 юаней/170 долл. США) от средних прямых затрат пациента (8 416 юаней/1 332 долл. США). В районе, где предоставлялась помощь, повышение доли завершения лечения на один процент обходилось дополнительно в 6 550 юаней (1 301 долл. США), а снижение доли неудачных исходов лечения на один процент обходилось дополнительно в 5 240 юаней (825 долл. США).

Заключение: В целом, предоставляемая финансовая помощь доказала свою эффективность в увеличении числа пациентов, завершивших лечение, и в сокращении числа неудачных исходов лечения среди больных

туберкулезом мигрантов в Шанхае. Полученные результаты показывают, что финансовая помощь может быть эффективно использована в качестве стратегии по улучшению ведения больных туберкулезом мигрантов в крупных городах Китая; этот подход может быть применим в аналогичных ситуациях на международном уровне.

Translated from English version into Russian by stasishe, through

TRANSLATORS
WITHOUT BORDERS

Proporcionar incentivos financieros a los migrantes rurales-urbanos que padecen TB en Shanghái: un estudio de intervención

Xiaolin Wei, Guanyang Zou, Jia Yin, John Walley, Huaixia Yang, Merav Kliner, Jian Mei

Resumen

Antecedentes: Los asuntos financieros son una barrera importante para los migrantes rurales-urbanos que quieren acceder a los servicios de atención de TB en China. Este trabajo informa sobre la eficacia de proporcionar incentivos financieros para el cumplimiento del tratamiento de los pacientes con TB, especialmente los migrantes pobres. Informa sobre las tasas de incumplimiento en un distrito de Shanghái, el efecto de los incentivos financieros en términos de reducción costo-paciente con TB y la relación gradual costo-eficacia de la intervención.

Resultados: Se registraron 90 y 93 pacientes migrantes con TB en las zonas de intervención y de control, respectivamente. Las tasas de cumplimiento del tratamiento de TB mejoraron significativamente en un 11% (de 78 a 89%) en la zona de intervención, en comparación con un aumento del 3% (de 73 a 76%) en la zona de control (P = 0,03). Las tasas de incumplimiento disminuyeron significativamente en un 11% (del 22 al 11%) en la zona de intervención, en comparación con el 1% (de 24 a 23%) en la zona de control (P = 0,03). La subvención financiera (RMB 1080 / USD 170) representó el 13% del costo directo promedio de los pacientes (RMB 8416 / USD 1332), en la zona de intervención. Cada uno por ciento de aumento, al cumplimiento del tratamiento, tiene un costo adicional de RMB 6550 (USD 1301) - o cada uno por ciento de reducción del incumplimiento tiene un costo adicional de RMB 5240 (USD 825), en la zona de intervención.

Conclusión: En general, los incentivos financieros han demostrado ser eficaces para mejorar el cumplimiento del tratamiento y la reducción de las tasas de incumplimiento entre los pacientes migrantes con TB en Shanghái. Los resultados sugieren que los incentivos financieros pueden utilizarse eficazmente como una estrategia para mejorar la gestión de casos en pacientes migrantes con TB en las grandes ciudades de China, lo que puede ser aplicable a escenarios internacionales similares.

Translated from English version into Spanish by Rosina Peixoto, through

TRANSLATORS
WITHOUT BORDERS
